# Prompt-to-Pill: Multi-Agent Drug Discovery and Clinical Simulation Pipeline

**DOI:** 10.1093/bioadv/vbaf323

**Published:** 2025-12-23

**Authors:** Ivana Vichentijevikj, Kostadin Mishev, Monika Simjanoska Misheva

**Affiliations:** iReason LLC, Skopje 1000, North Macedonia; Faculty of Computer Science and Engineering, Ss. Cyril and Methodius University, Skopje 1000, North Macedonia; Faculty of Computer Science and Engineering, Ss. Cyril and Methodius University, Skopje 1000, North Macedonia

## Abstract

**Summary:**

This study presents a proof-of-concept, comprehensive, modular framework for AI-driven drug discovery (DD) and clinical trial simulation, spanning from target identification to virtual patient recruitment. Synthesized from a systematic analysis of 51 large language model (LLM)-based systems, the proposed *Prompt-to-Pill* architecture and corresponding implementation leverages a multi-agent system (MAS) divided into DD, preclinical and clinical phases, coordinated by a central *Orchestrator*. Each phase comprises specialized LLM for molecular generation, toxicity screening, docking, trial design, and patient matching. To demonstrate the full pipeline in practice, the well-characterized target Dipeptidyl Peptidase 4 (DPP4) was selected as a representative use case. The process begins with generative molecule creation and proceeds through ADMET (Absorption, Distribution, Metabolism, Excretion, and Toxicity) evaluation, structure-based docking, and lead optimization. Clinical-phase agents then simulate trial generation, patient eligibility screening using electronic health records (EHRs), and predict trial outcomes. By tightly integrating generative, predictive, and retrieval-based LLM components, this architecture bridges drug discovery and preclinical phase with virtual clinical development, offering a demonstration of how LLM-based agents can operationalize the drug development workflow *in silico*.

**Availability and implementation:**

The implementation and code are available at: https://github.com/ChatMED/Prompt-to-Pill.

## 1 Introduction

The ability of large language model (LLMs) to learn from massive datasets and adapt to diverse inputs provides unprecedented capabilities that surpass traditional methods. Their use accelerates decision-making and reduces experimental costs across the development pipeline (Oniani *et al.* 2024), as evidenced by applications ranging from generating novel molecular structures (Li *et al.* 2023, Sheikholeslami *et al.* 2025) to predicting pharmacokinetics and toxicity (Liu *et al.* 2024, Cai *et al.* 2025), simulating clinical trials (Xu *et al.* 2025), and optimizing patient-trial matching (Lin *et al.* 2024, Datta *et al.* 2025).

The integration of LLMs into drug development pipelines has gained notable traction, especially across preclinical phases.

Gao *et al.* proposed a domain-guided multi-agent system (MAS) for reliable drug-target interaction (DTI) prediction, using a debate-based ensemble of LLMs. The framework partitions the DTI task into protein sequence understanding, drug structure analysis, and binding inference, handled by dedicated agents. Evaluation was conducted on the BindingDB dataset, showing improvements in both accuracy and prediction consistency compared to single-LLM baselines. The system integrates *GPT-4o*, *LLaMA-3*, and *GLM-4-Plus* (Gao *et al.* 2024).

Lee *et al.* developed *CLADD*, a retrieval-augmented MAS addressing multiple DD tasks. CLADD includes specialized teams for molecular annotation, knowledge graph querying, and prediction synthesis. Evaluations spanned property-specific captioning (BBBP, SIDER, ClinTox, BACE), target identification (DrugBank, KIBA), and toxicity classification. All agents were instantiated with *GPT-4o-mini*, showcasing the utility of general-purpose models when combined with structured Retrieval-augmented generation (RAG) mechanisms (Lee *et al.* 2025).

Song *et al.* presented *PharmaSwarm*, a hypothesis-driven agent swarm for therapeutic target and compound identification. The architecture orchestrates three specialized agents (*Terrain2Drug*, *Market2Drug*, *Paper2Drug*) and a central evaluator, all integrated via a shared memory and tool-augmented validation layer. Case studies included idiopathic pulmonary fibrosis and triple-negative breast cancer, combining omics analysis, literature mining, and market signals. Agents were powered by *GPT-4*, *Gemini 2.5*, and *TxGemma* (Song *et al.* 2025).

Yang *et al.* proposed *DrugMCTS*, a novel multi-agent drug repurposing system that incorporates Monte Carlo Tree Search (MCTS) with structured agent workflows. Using *Qwen2.5-7B-Instruct* for all agents, the system conducts iterative reasoning across molecule retrieval, analysis, filtering, and protein matching. The framework was benchmarked on DrugBank and KIBA, achieving up to 55.34% recall. A case study involving Equol and CXCR3 showed successful prediction of interaction, supported by AutoDock Vina simulations with a binding score of −8.4 kcal/mol (Yang *et al.* 2025).

Inoue *et al.* introduced *DrugAgent*, an explainable multi-agent reasoning system for drug repurposing. Their architecture coordinates agents handling knowledge graph queries, machine learning scoring, and biomedical literature summarization. Evaluation on a kinase inhibitor dataset revealed strong interpretability and modularity. Detailed ablation studies confirmed that each agent contributes distinctly to the performance. The system employed *GPT-4o*, *o3-mini*, and *GPT-4o-mini*, and the full pipeline is available open-source (Inoue *et al.* 2025). Among the surveyed systems, only DrugAgent provides a publicly accessible implementation (https://anonymous.4open.science/r/DrugAgent-B2EA).

None of the described MASs engages with clinical trial simulation, real-world evidence (RWE), or electronic health records (EHRs), thereby limiting their applicability to the preclinical stage of drug development.

This paper introduces *Prompt-to-Pill*, a unified multi-agent framework build on a systematic analysis of 51 LLM-based studies published between 2022 and 2025. The architecture integrates specialized LLM agents for molecule generation, docking, property prediction, trial construction, patient matching, and outcome forecasting through a central Orchestrator. Unlike prior frameworks confined to molecule-level reasoning, *Prompt-to-Pill* provides a proof-of-concept prototype from molecular ideation to virtual trial execution, demonstrating how modular LLM agents can operate synergistically within a closed-loop DD and development ecosystem. A complete implementation of the pipeline is available at GitHub (https://github.com/ChatMED/Prompt-to-Pill).

## 2 Methods

**Figure 1.**
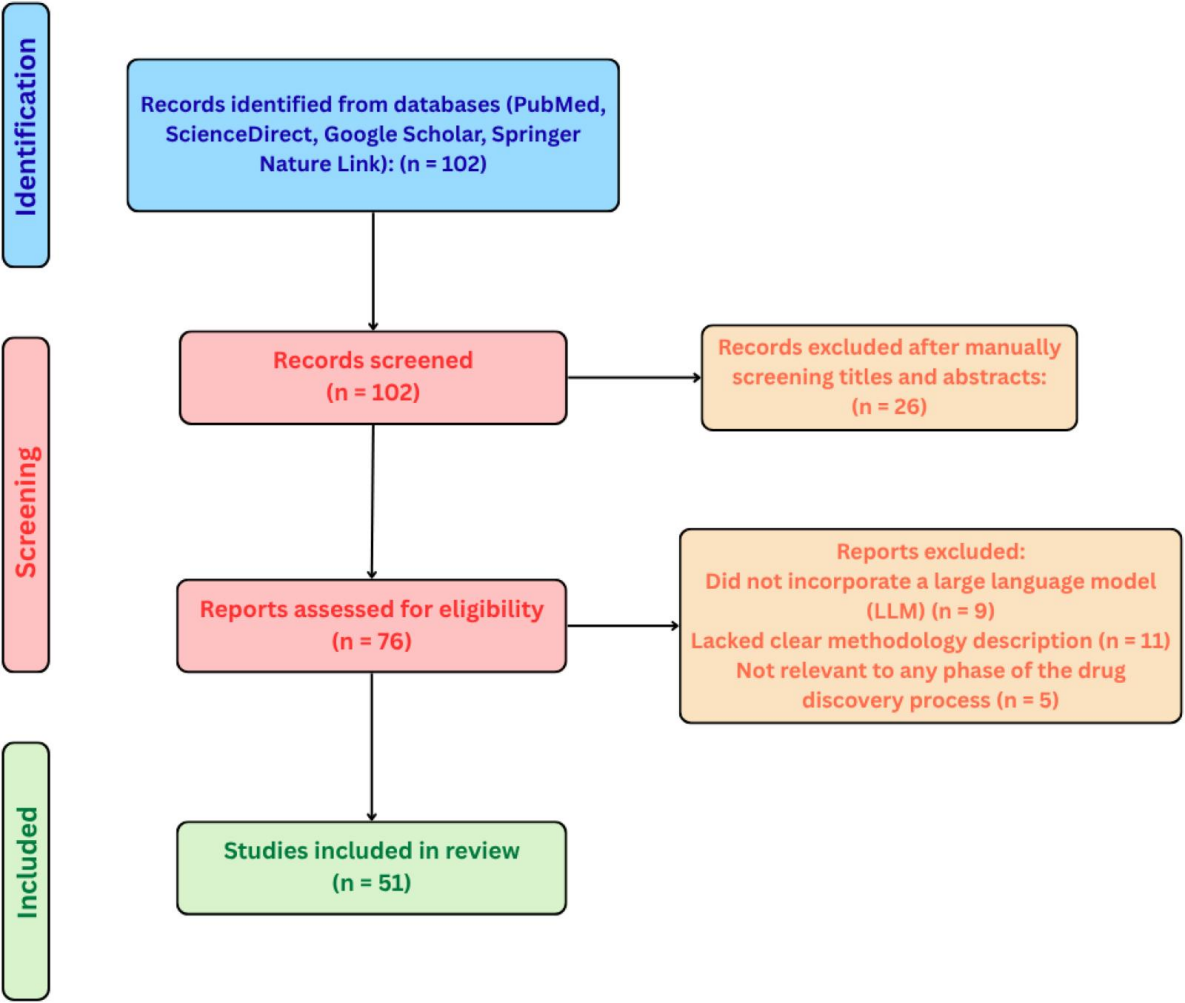
PRISMA-based selection process.

The systematic review was conducted in accordance with the Preferred Reporting Items for Systematic Reviews and Meta-Analyses (PRISMA) guidelines. The PRISMA framework was employed to ensure transparency, methodological rigor, and reproducibility in identifying, screening, and synthesizing eligible studies. A structured multi-stage review process was followed, encompassing database search, eligibility screening, full-text assessment, and data extraction. The complete selection workflow is detailed in the accompanying PRISMA flow diagram depicted in Fig. 1.

### 2.1 Information sources and search strategy

A structured and comprehensive literature search was conducted to identify and evaluate LLM-based approaches applied in drug design and discovery. The search was conducted between 1 May and 15 June 2025, across PubMed, ScienceDirect, Google Scholar, and Springer Nature Link. The search covered the publication period 2022–5.

Search queries with predefined Boolean combinations captured studies across all DD stages. Representative search strings included: *“large language models” AND (“target identification” OR “binding site prediction”), “large language models” AND (“molecule generation” OR “de novo molecule generation”), “large language models” AND (“clinical trial design” OR “eligibility criteria extraction” OR “trial outcome prediction”), “retrieval-augmented generation” AND (“drug discovery” OR “clinical trials”), “large language models” AND (“patient recruitment” OR “clinical trial matching”)*. These terms were selected to align with a conceptual pipeline spanning DD, preclinical and clinical phases of pharmaceutical development.

### 2.2 Study selection process

Two reviewers independently screened the titles and abstracts of all retrieved records. Full-text reviews were then performed to assess eligibility based on the predefined inclusion and exclusion criteria.

The inclusion criteria were defined as follows: open-source studies written in English; publications or preprints published between 2022 and 2025; research incorporating LLMs for drug development tasks with clearly defined input–output structure, functional purpose, and workflow integration potential; and studies relevant to at least one stage of the DD or clinical trial process.

The exclusion criteria were: articles not written in English; studies lacking a clear methodological or architectural description; studies that are not publicly accessible; and research not directly applicable to any stage of drug development.

The PRISMA flow diagram in Fig. 1 details the number of records identified, screened, excluded (with reasons), and finally taken into consideration for building the Prompt-to-Pill pipeline.

### 2.3 Data extraction and synthesis

For each included study, detailed metadata were manually extracted into structured tables, one for preclinical models and DD and another for clinical applications. Metadata fields were designed to support both technical evaluation and contextual information from each source as follows:

- **Bibliographic**: Authors, Year, Title, DOI.
- **Technical**: Base model (e.g. GPT-4, BioGPT), Task Type, RAG usage, Evaluation Metrics, Datasets.
- **Reproducibility**: GitHub/Hugging Face links, Input/Output examples.
- **Contextual**: Task Narrative, Clinical Trial Phase (I–IV), Abstract Summary.

Extracted data were then synthesized by stage as needed for the drug development pipeline. Studies were profiled and compared across multiple dimensions including application scope, base architecture, task type, and dataset diversity. The complete metadata tables containing all reviewed studies are provided in the section Data Availability. This structured comparison informed the construction of the Prompt-to-Pill multi-agent framework introduced later in the paper.

## 3 Methodology

### 3.1 Prompt-to-pill architecture foundation

The systematic review of 51 studies (2022–5) shows a sharp growth in research, peaking in 2024 (14 preclinical/DD, 8 clinical), with 17 in 2025, and fewer in 2022 (4) and 2023 (8).

Preclinical/DD studies mainly used generative LLMs such as *LLaMA* and *GPT-2* for creative molecular tasks on open datasets (TDC, DrugBank). Models like *DrugGen* (Sheikholeslami *et al.* 2025) and *DrugGPT* (Li *et al.* 2023) generate SMILES from protein sequences, 3D structures, or text, while others introduce spatial constraints [*3DSMILES-GPT* (Wang *et al.* 2025b)] or RNA design [*GenerRNA* (Zhao *et al.* 2024)]. *DrugAssist* (Ye *et al.* 2023) extends this process with prompt-based molecule optimization, refining compounds to improve pharmacological properties. LLMs also support ADMET (Absorption, Distribution, Metabolism, Excretion, and Toxicity) prediction, synthesis feasibility, and reactivity analysis (Chaves *et al.* 2024, Cai *et al.* 2025, Wang *et al.* 2025a), as well as biological interaction modeling and drug repurposing (Edwards *et al.* 2023, Beasley *et al.* 2025, Li *et al.* 2025, Schmitt *et al.* 2025).

Clinical studies, in contrast, rely on discriminative or hybrid models such as *GPT-4* and *BioBERT*, often trained on structured data (e.g. ClinicalTrials.gov). About half of the 19 clinical papers propose cross-phase models addressing patient selection, outcome prediction, and document generation. LLMs assist in patient-trial matching (Lin *et al.* 2024, Datta *et al.* 2025), trial simulation (Reinisch *et al.* 2024, Wang *et al.* 2024, Xu *et al.* 2025), and pharmacovigilance through tools like *AskFDALabel* and *DAEDRA* (von Csefalvay 2024, Wu *et al.* 2025), occasionally enhanced with RAG pipelines for context-aware text generation (Markey *et al.* 2025, Painter *et al.* 2025).

RAG methods showed limited adoption despite their potential for complex reasoning tasks. As shown in Table 1, most studies provided input examples but fewer included output data or reproducible code, underscoring the need for transparency and standardized evaluation.

**Table 1.** Availability of I/O Examples, RAG Usage, and code links in studies.

Category	Preclinical/DD			Clinical		
	Yes	No	N/A	Yes	No	N/A
GitHub/HF link	20	12	–	9	10	–
Input examples	17	2	1	6	2	1
Output examples	6	13	1	1	7	1
RAG usage	3	29	–	6	13	–

This analysis informed the design of the proposed Prompt-to-Pill architecture, implemented using the AutoGen (Wu *et al.* 2023) framework for scalable multi-agent AI systems. Each agent is adapted from rigorously evaluated domain models, with key performance metrics summarized in Table 2.

**Table 2.** Evaluation metrics of the core agents used in the clinical workflow.^a^

Citation	Agent	Dataset/Task	Metric(s)	Value(s)	Type
Sheikholeslami *et al.* (2025)	Molecule Generation (Druggen)	–	Validity, Novelty, Diversity	99.9%, 41.88%, 60.32%	Generation
Cai *et al.* (2025)	Property Prediction (ChemFM)	Drug Oral Bioavailability	ROC-AUC	0.715 ± 0.011	Classification
		BBB	ROC-AUC	0.908 ± 0.010	Classification
		Drug Half-Life Duration	Spearman	0.551 ± 0.020	Regression
		Drug Mutagenicity	ROC-AUC	0.854 ± 0.007	Classification
		Clearance Hepatocyte	Spearman	0.495 ± 0.030	Regression
		Clearance Microsome	Spearman	0.611 ± 0.016	Regression
		DILI	ROC-AUC	0.920 ± 0.012	Classification
		hERG Channel Blockage	ROC-AUC	0.848 ± 0.009	Classification
		Drug Acute Toxicity	MAE	0.541 ± 0.015	Regression
		PPBR	MAE	7.505 ± 0.073	Regression
		P-glycoprotein Inhibition	ROC-AUC	0.931 ± 0.003	Classification
		Drug Aqueous Solubility	MAE	0.725 ± 0.011	Regression
		VDss	Spearman	0.662 ± 0.013	Regression
		CYP2C9 Inhibition	PRC-AUC	0.788 ± 0.005	Classification
		CYP3A4 Inhibition	PRC-AUC	0.878 ± 0.003	Classification
		CYP2C9 Substrate	PRC-AUC	0.414 ± 0.027	Classification
		CYP2D6 Inhibition	PRC-AUC	0.704 ± 0.003	Classification
		CYP2D6 Substrate	PRC-AUC	0.739 ± 0.024	Classification
		Human IA	ROC-AUC	0.984 ± 0.004	Classification
		CYP3A4 Substrate	ROC-AUC	0.654 ± 0.022	Classification
		Drug Permeability	MAE	0.322 ± 0.026	Regression
Wang *et al.* (2024)	Trial Outcome Prediction(Meditab)	Phase I Trials	AUROC, PRAUC	0.699, 0.726	Classification
		Phase II Trials	AUROC, PRAUC	0.706, 0.733	Classification
		Phase III Trials	AUROC, PRAUC	0.734, 0.881	Classification
Ye *et al.* (2023)	Molecule Optimization (DrugAssist)	–	Solubility, BBBP, All, Valid rate, Similarity	0.74, 0.80, 0.62, 0.98, 0.69	Generation
Lin *et al.* (2024)	PatientMatching (Panacea)	SIGIR	BACC, F1, R, P	0.43, 0.57, 0.52, 0.66	Classification
		TREC 2021	BACC, F1, R, P	0.47, 0.58, 0.54, 0.69	Classification
	TrialDesign (Panacea)	Criteria	BLEU, ROUGE, CR	0.24, 0.44, 0.68	Generation
		Arms	BLEU, ROUGE, CR	0.28, 0.50, 0.61	Generation
		Outcome	BLEU, ROUGE, CR	0.31, 0.51, 0.55	Generation

a *Source*: Metrics are based on results reported in the original publications. ROC-AUC/AUROC: area under the receiver operating characteristic curve; MAE: mean absolute error; PRC-AUC/PRAUC: area under precision-recall curve; BACC: balanced accuracy; R: recall; P: precision; F1: F1 score; BLEU: bilingual evaluation understudy; ROUGE: recall-oriented understudy for Gisting evaluation; CR: clinical relevance.

The datasets listed in Table 2 implicitly define the applicability domains (ADs) of the models integrated into the pipeline. For example, ChemFM’s ADME and toxicity predictors are trained on specific benchmarking collections of drug-like compounds, while Panacea and MediTab operate within the disease areas and trial structures represented in CT.gov, SIGIR, and TREC. Because Prompt-to-Pill connects these components sequentially, the effective AD of the full system corresponds to the intersection of all model-specific ADs.

### 3.2 The Prompt-to-Pill multi-agent pipeline

**Figure 2.**
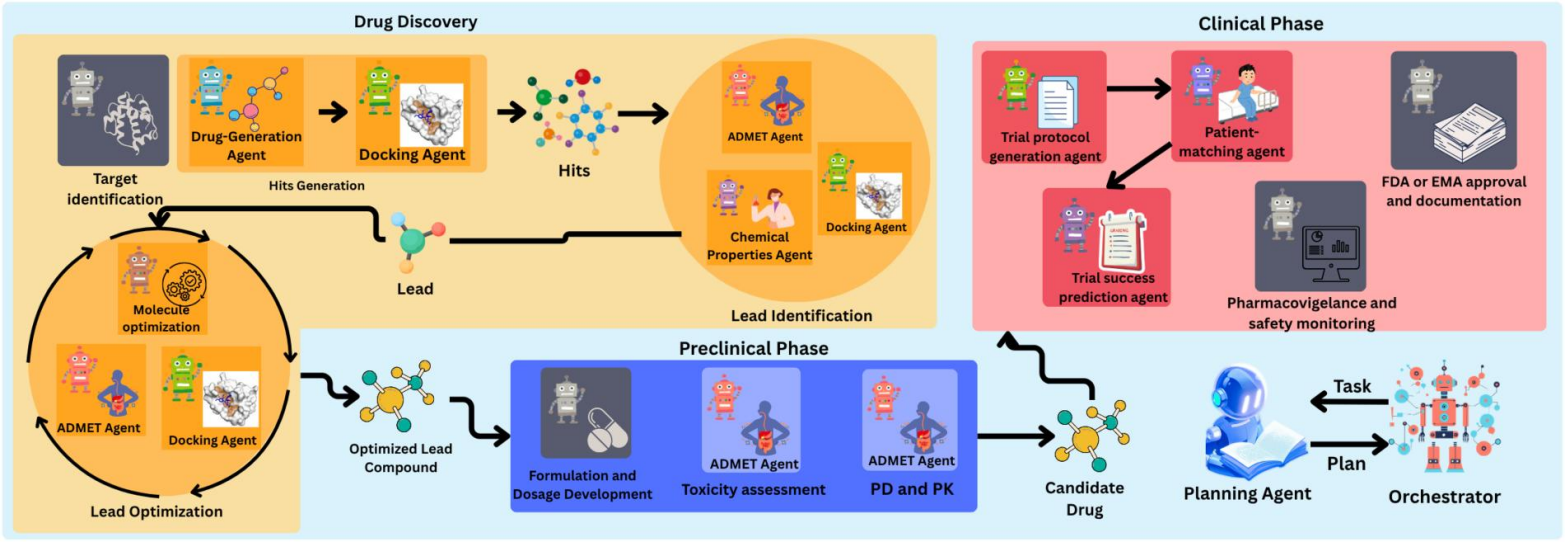
Prompt-to-pill multi-agent architecture.

Constructed from the models identified and reviewed in this study, a comprehensive AI-driven pipeline for DD and clinical trial simulation is presented in Fig. 2, structured into three main phases: DD Agents, Preclinical Agents and Clinical Agents, coordinated by a central *Orchestrator*, assisted by a *Planning Agent*. The workflow is task-driven, dynamically selecting the appropriate agent and its tools according to the requirements of the given task.

In our scenario, we demonstrate this process by focusing on the development of drug candidates for the DPP4 protein target (UniProt ID: P27487). For this drug development task, the pipeline begins with **Drug Discovery Agents**. Here we have three subgroups of agents: Hits Generation, Leads Identification, and Lead Optimization.

**Figure 3.**
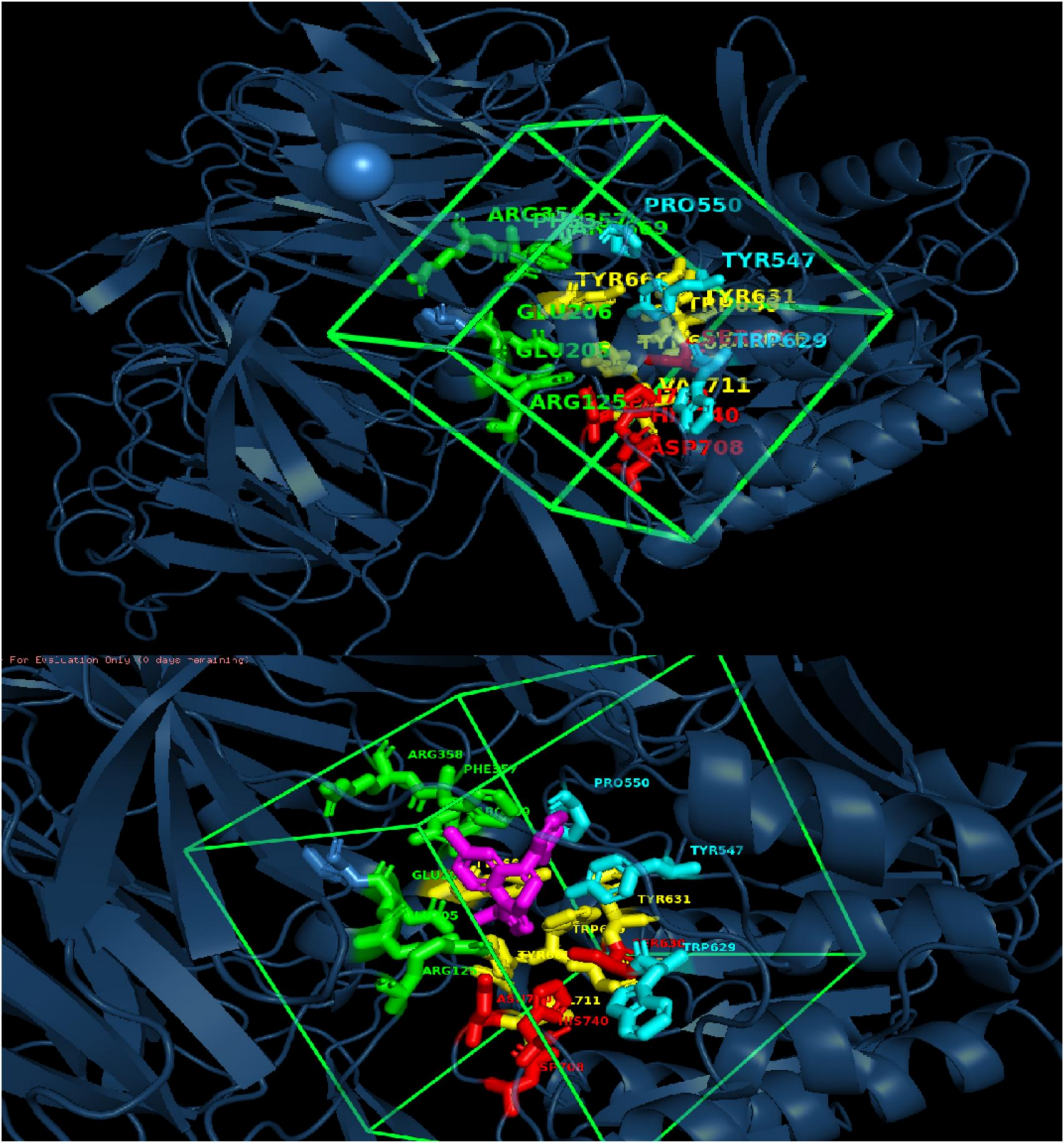
Docking visualization of DPP4 (PDB ID: 2QT9) showing the predicted binding pocket (green grid box; center = 37.87, 49.09, 36.58; edge = 25.02 Å) and the docked ligand (magenta; SMILES: OB(O)c1nnc2n1-c1ccc(Cl)cc1C(c1ccccc1F)=NC2). Pocket side chains are shown as colored sticks (colors for visual separation only) and correspond to validated binding residues ARG125, GLU205, GLU206, TYR547, TYR631, SER630, HIS740, and ASN710 (Mathur *et al.* 2023).

The workflow begins with *Hits Generation*, where the *Drug-Generation Agent*, based on the DrugGen framework (Sheikholeslami *et al.* 2025), produces a set of candidate SMILES sequences. Then the generated SMILES are docked against the target with *Docking Agent*. The Docking Agent is responsible for evaluating the binding affinity of generated molecules against the target protein. Retrieves the target structure from the Protein Data Bank (Berman *et al.* 2000) or defaults to AlphaFold models (Jumper *et al.* 2021) when no experimental structure is available. Candidate SMILES from the *Drug-Generation Agent* are converted into 3D conformations using RDKit (https://www.rdkit.org). Binding pockets are predicted with P2Rank (Krivák and Hoksza 2018) (P2Rank success rates: 72.0% Top-n, 78.3% Top-(n+2) on COACH420; 68.6% Top-n, 74.0% Top-(n+2) on HOLO4K), and the highest-ranked pocket defines the docking box, whose coordinates are extracted from the P2Rank output and expanded with a fixed padding margin. With receptor and ligand prepared, AutoDock Vina (v1.1.2) performs docking within the predicted pocket, generating 20 poses ranked by affinity. Using this approach, we achieved RMSD lower than 2 Å in 86.59% of cases and a mean RMSD of 1.16 Å on the Astex dataset. The docking setup and visualization, including binding sites, grid box, and ligand, are shown in Fig. 3.

Following the generation and docking of hits, the workflow progresses to the *Lead Identification* stage. The *Chemical Properties Agent* calculates key physicochemical descriptors (molecular weight, logP, TPSA, hydrogen bond donors and acceptors, rotatable bonds, QED, etc.) using RDkit driven tools. Molecules are filtered according to Lipinski’s Rule of Five (HBD ⩽ 5, HBA ⩽ 10, MW ⩽ 500, logP ⩽ 5) (Lipinski *et al.* 2001) and Veber’s rules (RotB ⩽ 10, TPSA ⩽ 140 Å) (Veber *et al.* 2002), ensuring that only drug-like compounds advance. In parallel, the *ADMET Properties Agent*, using ChemFM (Cai *et al.* 2025) framework, is also invoked at this stage to provide an early assessment of ADMET. Properties that this agent can predict are presented in 2. Compound that show the most favorable docking, pass physicochemical filters, and exhibit acceptable ADMET predictions are prioritized as lead.

Next is *Lead Optimizations* stage. This stage focuses on optimizing the chosen molecule to enhance its pharmacological profile while preserving strong binding affinity to the DPP4 target. The *Molecule Optimization Agent*, based on DrugAssist (Ye *et al.* 2023), iteratively modifies the structure to enhance bioavailability, solubility, and safety. Each optimized variant is re-evaluated by the *ADMET Properties Agent* and *Docking Agent*, and this loop continues until optimal properties are achieved.

The optimized compound with properties serve entry point into the **Preclinical Phase**, where the optimized candidate undergoes systematic pharmacokinetic and toxicity profiling using ADMET Agent’s tools. Once these evaluations are completed, the workflow is shifted into the **Clinical Phase** for trial simulation.

In the Clinical Phase, the *Trial Generation Agent* constructs a trial protocol tailored to the compound and disease driven by *Panacea* model for criteria, arms and outcomes prediction. This protocol is parsed into structured data and passed to the *Patient-Matching Agent*, which also employs the *Panacea* model (Lin *et al.* 2024) to evaluate patient EHR descriptions and identify candidates who meet the trial’s inclusion and exclusion criteria. The agent returns number of matched patients in the final report, and a set of matched patient IDs. These identifiers are saved to a file and the total number of matched patients is computed and included in the final trial report. Subsequently, the *Trial Outcome Prediction Agent* uses *MediTab* (Wang *et al.* 2024) to estimate the probability that the proposed trial will succeed, given its protocol structure. In line with the original MediTab formulation (Wang *et al.* 2024), this module operates on trial-level metadata and text and learns patterns from historical ClinicalTrials.gov and HINT benchmarks.

Finally, the matched patient data, drug properties, and trial design are provided to the *Orchestrator*, which aggregates all outputs into a structured report.

**Figure 4.**
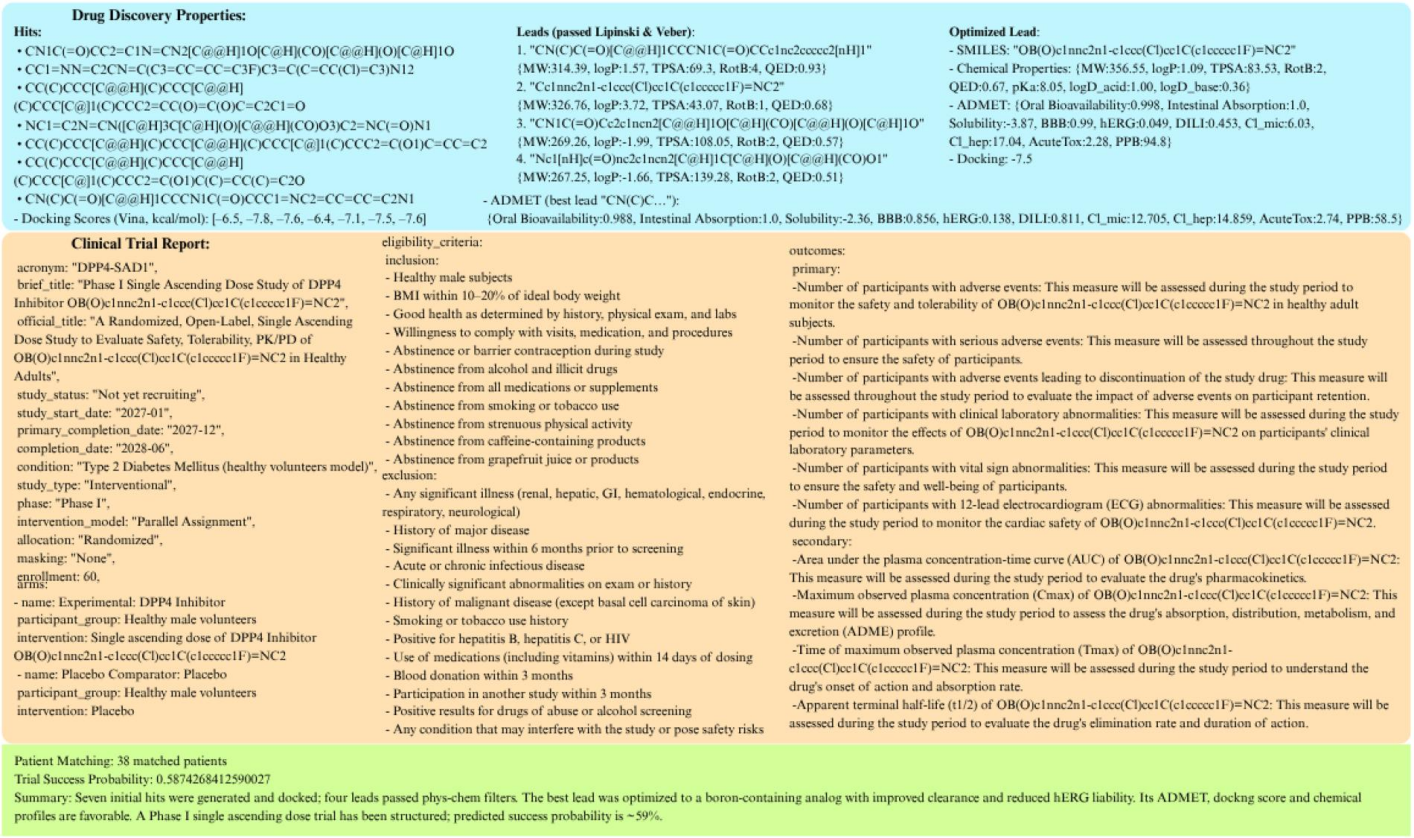
Prompt-to-Pill’s I/O example for Task: “Simulate drug development for DPP4 (P27487) with patients on/path/to/patients.xml”. “Trial Success Probability” correspond to MediTab’s predicted likelihood of clinical trial success based on protocol text and structured trial metadata (e.g. phase, condition, enrollment, arms, outcomes), and do not represent estimates of the underlying drug’s biological efficacy.

The input and output format for the Dipeptidyl peptidase 4 (DPP4) target are shown in Fig. 4.

## 4 Limitations and future work

Prompt-to-Pill is designed as a research-oriented, hypothesis-generation framework intended for use exclusively by trained professionals such as bioinformaticians, medicinal chemists, pharmacologists, and clinicians. The system is not intended for clinical or regulatory decision-making. Instead, its outputs serve as exploratory insights that must be experimentally or clinically validated before any real-world application. The framework aims to support early ideation, academic research, and computational prototyping. While the proposed Prompt-to-Pill pipeline offers a structured, automated approach to drug discovery and clinical simulation, several limitations remain.

First, as shown in Fig. 2, some agents remain conceptual placeholders (highlighted in grey), like *Target Identification Agent*, the *Formulation and Dosage Development Agent*, *FDA or EMA approval and documentation* and the *Pharmacovigilance and Safety Monitoring Agent*. Although we identified related approaches in the literature, most lack accessible implementations or compatible I/O interfaces, preventing integration. Bridging this gap remains a key direction for future work.

Second, the *Orchestrator* is currently implemented using OpenAI’s o4-mini model, which has been shown to perform strongly in medical reasoning and biomedical tasks Arora *et al.* (2025). However, in the trial generation phase, its role is used to producing structured protocol fields such as: study documents, brief summary, acronym, brief title, official title, study status, study start date, primary completion date, completion date, condition, study type, phase, intervention model, allocation, masking, and enrollment. While useful for structuring and simulating trial protocols, these outputs cannot substitute for expert-driven trial design.

Third, each component model operates within the applicability domain (AD) of its training data, and the pipeline therefore inherits the intersection of all ADs. Predictions involving molecules, trial structures, or patient populations far from these distributions should be interpreted as exploratory, not definitive.

This study also presents only a single-target case (DPP-4, P27487), demonstrating feasibility but not generalizability. Future work will extend the framework to multiple targets and diseases for broader validation.

Future work will focus on completing missing agents, enlarging the AD of existing components, and performing multi-disease, multi-target validation studies.

## 5 Discussion

While LLMs have opened transformative opportunities in drug discovery and clinical research, realizing their full potential requires addressing key challenges in transparency, evaluation consistency, and reproducibility.

A key limitation is the limited reasoning ability of current models. In biomedical contexts, correctness alone is insufficient—decisions must be grounded in clear, interpretable reasoning that experts can verify. To address this, several recent models have introduced mechanisms to make reasoning more explicit. These include retrieval-augmented generation (Feng *et al.* 2025, Wang *et al.* 2025a, Xu *et al.* 2025), instruction-tuned multitask learning (Liu *et al.* 2024, Ma *et al.* 2024), and multi-hop rationale generation (Wang *et al.* 2023, Feng *et al.* 2025). Such approaches represent important progress toward interpretability. However, without standardized frameworks to assess reasoning quality or consistency, trust in LLM-driven biomedical insights remains limited.

Another major challenge in LLM-based DD is the inconsistency in evaluation protocols across model types. Generative models are assessed using metrics like validity, docking scores, or QED (Zhao *et al.* 2024, Sheikholeslami *et al.* 2025, Wang *et al.* 2025b), while discriminative models report AUROC or F1 scores (Liu *et al.* 2024, Ma *et al.* 2024, Wang *et al.* 2025a), yet differ in datasets and thresholds. Knowledge-retrieval and reasoning systems often rely on qualitative outputs without standardized measures (Wang *et al.* 2023, Feng *et al.* 2025). This fragmentation hinders comparability and progress. To address this, the field urgently needs task-specific, model-type-sensitive benchmarks.

Reproducibility and transparency also remain persistent issues. Many studies lack public access to code, models, or I/O examples, and when repositories exist, documentation is often incomplete. This fragmentation limits cumulative progress and undermines trust.

These models are the future of drug development, but there is still much work to be done. The path forward requires not only better models, but better systems around the models. This includes standardized evaluations, transparent documentation, expert-guided development, and thoughtful regulation. Only by meeting these unmet needs can we ensure that LLMs evolve from experimental tools to trusted agents in the future of biomedical discovery.

## 6 Ethical and regulatory considerations

As highlighted in recent discussions on responsible biomedical AI deployment (Tang *et al.* 2025), LLM-based systems in safety-critical domains such as clinical trial design raise central concerns around transparency, explainability, bias mitigation, and the risk of over-reliance on unvalidated outputs. The European Union’s Artificial Intelligence Act (2024) explicitly designates healthcare AI as “high-risk” (Recital 58; Annex III), requiring safeguards such as traceability, human oversight, and fundamental rights impact assessments. The authors have recently published their work on AI Act compliance within the MyHealth@EU framework (Simjanoska Misheva *et al.* 2025), demonstrating strong ethical responsibility in advancing AI use within sensitive healthcare environments. Their tutorial addresses the dual-compliance challenge of embedding AI Act safeguards (transparency, provenance, robustness) while meeting MyHealth@EU interoperability requirements, showing how AI metadata can be integrated into HL7 (Health Level Seven) CDA (Clinical Document Architecture) and FHIR (Fast Healthcare Interoperability Resources) messages without disrupting existing standards. The goal is not to bypass current guidelines but to ease clinicians’ workload, strengthen trust in AI-assisted decisions, and ensure that compliance and safety are engineered into systems from the outset. In this context, the present pipeline is strictly positioned as a research prototype and decision-support artifact, never as an automated tool for patient eligibility or therapeutic approval. By embedding governance mechanisms early and framing the work as proof-of-concept exploration, the approach contributes to the broader dialogue on trustworthy AI in DD while acknowledging the rigorous benchmarking, reproducibility, and expert oversight still required before clinical translation.

## 7 Conclusion

To illustrate practical integration, the Prompt-to-Pill multi-agent framework was proposed, uniting specialized LLM agents to automate decision-making across preclinical and clinical stages. This architecture showcases how coordinated LLM workflows can collaborate, iterate, and self-correct within a modular design. Crucially, the successful implementation of this architecture served simultaneously as a “limitation demonstrator,” significantly highlighting the applicability domain limitations and systemic challenges that must be overcome, thereby establishing a rigorous foundation upon which reliable hypothesis generation can be built.

Looking ahead, the progress of LLM-driven drug development will depend not only on more capable models but on robust evaluation protocols, transparent sharing, and clear regulatory standards. Addressing these challenges will allow LLMs not just to accelerate, but to redefine the future of drug development.

## Data Availability

The full implementation of the Prompt-to-Pill multi-agent DD and clinical simulation pipeline is available at the GitHub repository: https://github.com/ChatMED/Prompt-to-Pill. Table with the retrieved papers for this review is available at the following link: Supplementary Table 1.

## References

[vbaf323-B1] Arora RK, Wei J, Soskin Hicks R *et al*. HealthBench: evaluating large language models towards improved human health. arXiv, https://arxiv.org/abs/2505.08775, 2025, preprint: not peer reviewed.

[vbaf323-B2] Beasley J-MT, Schatz K, Ding E *et al*. TARRAGON: therapeutic target applicability ranking and retrieval-augmented generation over networks. bioRxiv. 10.1101/2025.04.19.649662, 2025, preprint: not peer reviewed.

[vbaf323-B3] Berman HM , Westbrook J, Feng Z et al The protein data bank. Nucleic Acids Res 2000;28:235–42. 10.1093/nar/28.1.235 10592235 PMC102472

[vbaf323-B4] Cai F, Zacour K, Zhu T *et al*. ChemFM as a scaling law guided foundation model pre-trained on informative chemicals. Commun Chem 2025. 10.1038/s42004-025-01793-8 PMC12764477 41413269

[vbaf323-B5] Chaves JMZ, Wang E, Tu T *et al*. Tx-LLM: a large language model for therapeutics. arXiv, https://arxiv.org/abs/2406.06316, 2024, preprint: not peer reviewed.

[vbaf323-B6] Datta S , Lee K, Huang L-C et al Patient2trial: from patient to participant in clinical trials using large language models. Inform Med Unlocked 2025;53:101615. 10.1016/j.imu.2025.101615.

[vbaf323-B7] Edwards C, Naik A, Khot T *et al*. SynerGPT: in-context learning for personalized drug synergy prediction and drug design. bioRxiv, 10.1101/2023.07.06.547759, 2023, preprint: not peer reviewed.

[vbaf323-B8] Feng Y , Wang J, He R et al A retrieval-augmented knowledge mining method with deep thinking LLMs for biomedical research and clinical support. GigaScience 2025;14:giaf109. 10.1093/gigascience/giaf109 40971592 PMC12448786

[vbaf323-B9] Gao B, Bai H, Zhang P. A multi-agent framework for reliable drug–target interaction prediction. Courseproposal,Advanced Machine Learning, Tsinghua University, 2024. https://openreview.net/forum?id=psdCKPT9wu

[vbaf323-B10] Inoue Y, Song T, Wang X *et al*. DrugAgent: multi-agent large language model-based reasoning for drug–target interaction prediction. arXiv, 10.48550/arXiv.2408.13378, 2025, preprint: not peer reviewed.

[vbaf323-B11] Jumper J , Evans R, Pritzel A et al Highly accurate protein structure prediction with alphafold. Nature 2021;596:583–9. 34265844 10.1038/s41586-021-03819-2 PMC8371605

[vbaf323-B12] Krivák R , Hoksza D. P2rank: machine learning based tool for rapid and accurate prediction of ligand binding sites from protein structure. J Cheminform 2018;10:39. 30109435 10.1186/s13321-018-0285-8 PMC6091426

[vbaf323-B13] Lee N, De Brouwer E, Hajiramezanali E *et al*. RAG-enhanced collaborative LLM agents for drug discovery. arXiv, https://arxiv.org/abs/2502.17506, 2025, preprint: not peer reviewed.

[vbaf323-B14] Li Y, Gao C, Song X *et al*. DrugGPT: a GPT-based strategy for designing potential ligands targeting specific proteins. bioRxiv, 10.1101/2023.06.29.543848, 2023, preprint: not peer reviewed.

[vbaf323-B15] Li Z, Chen XA, Jeon Y. GraPPI: a retrieve-divide-solve GraphRAG framework for large-scale protein–protein interaction exploration. arXiv, https://arxiv.org/abs/2501.16382, 2025, preprint: not peer reviewed.

[vbaf323-B16] Lin J, Xu H, Wang Z *et al*. Panacea: a foundation model for clinical trial search, summarization, design, and recruitment. medRxiv, 10.1101/2024.06.26.24309548, 2024, preprint: not peer reviewed.

[vbaf323-B17] Lipinski CA , Lombardo F, Dominy BW et al Experimental and computational approaches to estimate solubility and permeability in drug discovery and development settings. Adv Drug Deliv Rev 2001;46:3–26. 10.1016/s0169-409x(00)00129-0 11259830

[vbaf323-B18] Liu Y, Ding S, Zhou S *et al*. MolecularGPT: open large language model (LLM) for few-shot molecular property prediction. arXiv, https://arxiv.org/abs/2406.12950, 2024, preprint: not peer reviewed.

[vbaf323-B19] Ma T, Lin X, Li T *et al*. Y-Mol: a multiscale biomedical knowledge-guided large language model for drug development. arXiv, https://arxiv.org/abs/2410.11550, 2024, preprint: not peer reviewed.

[vbaf323-B20] Markey N , El-Mansouri I, Rensonnet G et al From rags to riches: utilizing large language models to write documents for clinical trials. Clin Trials 2025;22:626–31. 10.1177/17407745251320806 40013826 PMC12476469

[vbaf323-B21] Mathur V , Alam O, Siddiqui N et al Insight into structure activity relationship of dpp-4 inhibitors for development of antidiabetic agents. Molecules 2023;28:5860. 10.3390/molecules28155860 37570832 PMC10420935

[vbaf323-B22] Oniani D , Hilsman J, Zang C et al Emerging opportunities of using large language models for translation between drug molecules and indications. Sci Rep 2024;14:10738. 10.1038/s41598-024-61124-0. 38730226 PMC11087469

[vbaf323-B23] Painter JL , Chalamalasetti VR, Kassekert R et al Automating pharmacovigilance evidence generation: using large language models to produce context-aware structured query language. JAMIA Open 2025;8:ooaf003. 10.1093/jamiaopen/ooaf003 39926164 PMC11806702

[vbaf323-B24] Reinisch M, He J, Liao C *et al*. CTP-LLM: clinical trial phase transition prediction using large language models. In: *2024 IEEE International Conference on Bioinformatics and Biomedicine (BIBM)*, 2024, p. 3667–3672. 10.1109/BIBM62325.2024.10822746

[vbaf323-B25] Schmitt RA, Buelau K, Martin L *et al*. Biological database mining for LLM-driven Alzheimer’s disease drug repurposing. bioRxiv, 10.1101/2024.12.04.626255, 2025, preprint: not peer reviewed.

[vbaf323-B26] Sheikholeslami M , Mazrouei N, Gheisari Y et al Druggen enhances drug discovery with large language models and reinforcement learning. Sci Rep 2025;15:13445. 10.1038/s41598-025-98629-1 40251288 PMC12008224

[vbaf323-B27] Simjanoska Misheva M , Shahpaski D, Dobreva J et al Ai act compliance within the MyHealth@EU framework: a tutorial. J Med Internet Res 2025;27:e81184. 10.2196/81184 41213096 PMC12599979

[vbaf323-B28] Song K, Trotter A, Chen JY. LLM agent swarm for hypothesis-driven drug discovery. arXiv, https://arxiv.org/abs/2504.17967, 2025, preprint: not peer reviewed.

[vbaf323-B29] Tang X , Jin Q, Zhu K et al Risks of AI scientists: prioritizing safeguarding over autonomy. Nat Commun 2025;16:8317. 40968279 10.1038/s41467-025-63913-1 PMC12446425

[vbaf323-B30] Veber DF , Johnson SR, Cheng H-Y et al Molecular properties that influence the oral bioavailability of drug candidates. J Med Chem 2002;45:2615–23. 12036371 10.1021/jm020017n

[vbaf323-B31] von Csefalvay C. DAEDRA: a language model for predicting outcomes in passive pharmacovigilance reporting. arXiv, https://arxiv.org/abs/2402.10951, 2024, preprint: not peer reviewed.

[vbaf323-B32] Wang E, Schmidgall S, Jaeger PF *et al*. TxGemma: efficient and agentic LLMs for therapeutics. arXiv, https://arxiv.org/abs/2504.06196, 2025a, preprint: not peer reviewed.

[vbaf323-B33] Wang J , Luo H, Qin R et al 3dsmiles-gpt: 3d molecular pocket-based generation with token-only large language model. Chem Sci 2025b;16:637–48. 10.1039/D4SC06864E 39664804 PMC11629531

[vbaf323-B34] Wang Z, Xiao C, Sun J. AutoTrial: prompting language models for clinical trial design. In: *Proceedings of the 2023 Conference on Empirical Methods in Natural Language Processing (EMNLP)*, 2023, p. 12461–12472. 10.18653/v1/2023.emnlp-main.766

[vbaf323-B35] Wang Z, Gao C, Xiao C et al MediTab: scaling medical tabular data predictors via data consolidation, enrichment, and refinement. In: *Proceedings of the Thirty-Third International Joint Conference on Artificial Intelligence (IJCAI-24),* 2024, p. 6062–6070. 10.24963/ijcai.2024/670

[vbaf323-B36] Wu L , Fang H, Qu Y et al Leveraging FDA labeling documents and large language model to enhance annotation, profiling, and classification of drug adverse events with AskFDALabel. Drug Saf 2025;48:655–65. 10.1007/s40264-025-01520-1 39979771 PMC12098182

[vbaf323-B37] Wu Q, Bansal G, Zhang J et al AutoGen: enabling next-gen LLM applications via multi-agent conversation. arXiv, https://arxiv.org/abs/2308.08155, 2023, preprint: not peer reviewed.

[vbaf323-B38] Xu Z, Wu F, Lu Y et al Retrieval-reasoning large language model-based synthetic clinical trial generation. In: *Proceedings of the 16th ACM International Conference on Bioinformatics, Computational Biology, and Health Informatics*, 2025, p. 34. 10.1145/3765612.3767193

[vbaf323-B39] Yang Z, Wan Y, Yan S et al DrugMCTS: a drug repurposing framework combining multi-agent, RAG, and Monte Carlo tree search. arXiv, https://arxiv.org/abs/2507.07426, 2025, preprint: not peer reviewed.

[vbaf323-B40] Ye G, Cai X, Lai H et al DrugAssist: a large language model for molecule optimization. Brief Bioinform 2025;26:bbae693. 10.1093/bib/bbae693 PMC11697106 39751647

[vbaf323-B41] Zhao Y, Oono K, Takizawa H et al GenerRNA: a generative pre-trained language model for de novo RNA design. bioRxiv, 10.1101/2024.02.01.578496, 2024, preprint: not peer reviewed. PMC11444397 39352899

